# Spreading depolarization: A wave that precedes and drives cerebral ischemic cell death—Target for neuroprotection

**DOI:** 10.1177/0271678X261417186

**Published:** 2026-03-21

**Authors:** Umberto Pensato, Johanna M Ospel, Coline L Lemale, Jed A Hartings, Michele Romoli, Simona Sacco, Jens P Dreier

**Affiliations:** 1 Department of Biomedical Sciences, Humanitas University, Pieve Emanuele, Milan, Italy; 2 Department of Neurology, IRCCS Humanitas Research Hospital, Rozzano, Milan, Italy; 3 Department of Radiology, University of Calgary, Calgary, AB, Canada; 4 Center for Stroke Research Berlin, Charité Universitätsmedizin Berlin, Corporate Member of Freie Universität Berlin and Humboldt-Universität zu Berlin, Berlin, Germany; 5 Department of Experimental Neurology, Charité Universitätsmedizin Berlin, Corporate Member of Freie Universität Berlin and Humboldt-Universität zu Berlin, Berlin, Germany; 6 Department of Neurosurgery, University of Cincinnati College of Medicine, Cincinnati, OH, USA; 7 Department of Neurosciences, Bufalini Hospital, Cesena, Italy; 8 Department of Applied Clinical Sciences and Biotechnology, University of L’Aquila, L’Aquila, Italy; 9 Department of Neurology, Charité Universitätsmedizin Berlin, Corporate Member of Freie Universität Berlin and Humboldt-Universität zu Berlin, Berlin, Germany; 10 Bernstein Center for Computational Neuroscience Berlin, Berlin, Germany; 11 Einstein Center for Neurosciences Berlin, Berlin, Germany

**Keywords:** Ischemic stroke, cardiac arrest, cerebral ischemic injury, cerebral infarction, neuroprotection, spreading depression

## Abstract

Spreading depolarization (SD) is an all-or-none response of gray matter, evolutionarily conserved from insects to mammals and characterized by near-complete breakdown of transmembrane ion gradients and cytotoxic edema. In humans, SD has been recorded in diverse neurological conditions, including cerebral ischemia and hemorrhage, traumatic brain injury (TBI), and even during whole-brain death following cardiac arrest or during continued systemic circulation. During migraine aura, initial hyperemia accompanies short-lived and benign SD. SD under energy deficiency is also initially reversible. However, if intraneuronal Ca^2+^ increases persist for too long, cell death develops, characterized by a negative ultraslow potential. The same SD wave can be long-lasting and deadly in one place, but short and harmless in another. Energy depletion after arterial obstruction triggers SD within minutes, but SD can also arise in non-ischemic tissue and trigger vasoconstriction and ischemia as a result of an inverse hemodynamic response. Overall, SD may be both adaptive and maladaptive, depending on the tissue conditions. As final reversible stage before ischemic neuronal death, it is not only an orderly retreat from life, but can also allow a reboot. SD is a biomarker of neuronal injury during neurocritical care and a compelling yet complex therapeutic target for neuroprotection.

## Introduction

Spreading depolarization (SD) is a critical, all-or-none response in the central nervous system (CNS) characterized by an abrupt and near-complete breakdown of cell transmembrane ion gradients. It results in near-zero membrane potentials, swelling of neurons (cytotoxic edema), and extracellular ionic edema.^1–6^ SD was first identified in the 1940s through pioneering animal experiments conducted by the Brazilian physiologist Aristides Leão.^7^ In his original papers, he anticipated the pivotal role of SD in both migrainous aura and stroke within the human brain.^8,9^ Decades later, mounting evidence has largely validated his insights.^1,10–13^ While the headache community, after decades of controversy,^14^ has now widely recognized the pathophysiological role of SD in migrainous aura,^15,16^ awareness in stroke research that SD plays a fundamental role in the development of neuronal cell death is still in its nascent stages. Historically, the pathophysiology of cerebral infarction has been examined primarily through the lens of vasculature and perfusion. Acknowledging the role of SD requires broadening this perspective to encompass the neurovascular unit as a whole rather than focusing solely on the cerebrovascular tree. This expanded view highlights the multiple interactions and different players of this unit, including neurons, glia (astrocytes, microglia, oligodendrocytes), and vascular cells (endothelium, vascular smooth muscle cells, pericytes). All of these are crucially involved in the SD process, even though SD itself is, at its core, a neuronal mechanism.^17–19^

A key challenge for SD in the stroke field was that the phenomenon was repeatedly rediscovered in different contexts following its initial description by Leão. Over the years, it was given various names such as spreading depression, anoxic depolarization, and peri-infarct depolarization, creating the impression of distinct waves rather than a single, unified phenomenon.^20^ Yet it is now well established that these various terms were applied to describe different manifestations and properties of the same depolarization wave as it propagated through tissues with varying degrees of metabolic impairment. In 2017, the Co-Operative Studies on Brain Injury Depolarizations (COSBID) group addressed this issue by proposing a standardized nomenclature that provides a concise and logical framework to consistently describe and understand the phenomenon of SD across different diseases and conditions, from migraine to stroke to cardiac arrest.^1,21^ This framework captures the various SD manifestations related to different conditions of baseline perfusion and spontaneous activity that may even vary along the path of a single propagating SD wave. To assist the readers, we have included a glossary that follows this standardized terminology (Table 1).

**Table 1.** Glossary.

Commitment point	The critical moment when the metabolic supply/demand mismatch, along with the SD, has lasted long enough to cause irreversible ischemic damage of neurons. The time to reach the commitment point depends on the severity of the metabolic mismatch and the cumulative burden of SD
Ischemic core	Region of brain tissue that has already suffered irreversible injury and will progress to histological infarction, even if the tissue is reperfused. The development of the ischemic core is preceded by SD, which becomes terminal as it progresses
Ischemic penumbra	Region of brain tissue critically hypoperfused, that is, at risk of infarction, but which can still be saved with timely reperfusion. This area is associated with prolonged but transient SD
Negative ultraslow potential	Second component of the negative direct current shift, which immediately follows the initial, still reversible SD component and indicates the death of neurons during a terminal SD
Non-spreading depression	Simultaneously occurring depression of spontaneous brain activity in an ischemic brain region. This phenomenon is responsible for the immediate, multimodal clinical symptoms observed in patients with focal or global cerebral ischemia. SD occurs later in this tissue, but it will not lead to a patient percept since the brain activity is already suppressed
SD	An abrupt, nearly complete breakdown of cell transmembrane ion gradients that spreads through contiguous cerebral gray matter at a pace of 2–9 mm/min. SD can occur for no apparent reason (e.g. migrainous aura) or be provoked (e.g. ischemia). The duration of SD can vary from transient (self-resolving) to terminal (it lasted long enough to cause infarction)
Spreading depression	A wave of depression of cerebral spontaneous activity caused by SD. While the terms “SD” and “spreading depression” have sometimes been used interchangeably, they have distinctive meanings: spreading depression refers to the functional suppression of cerebral activity, whereas SD describes the sustained depolarization and near-complete disruption of the electrochemical transmembrane gradients with neuronal cytotoxic edema
Spreading hyperemia	Transient increase of cerebral blood flow triggered by SD in intact tissue
Spreading ischemia	Severe paradoxical decrease in cerebral blood flow that is triggered by SD in tissue with impaired neurovascular unit and prolongs the neuronal depolarization phase
Spreading oligemia	Still adequate, but reduced perfusion in intact tissue compared to the baseline level, which follows the repolarization after SD in otherwise healthy tissue

SD: spreading depolarization.

Here, we review the pathophysiology of SD, its clinical implications, and its critical role in neuronal death, especially in the dying of the whole brain and the development of ischemic infarction. Our main goal is to highlight some critical, clinically relevant features of SD, the knowledge of which may be of particular interest to those involved in developing neuroprotective strategies for stroke. In this context, it is important to underscore that there is increasing evidence that SD can have two possible roles, an adaptive and a maladaptive one, both of which should be considered for neuroprotective strategies.^11^

## SD physiology: The largest electrochemical phenomenon in the living CNS

SD is caused by a near-complete breakdown of the electrochemical barrier between the extracellular space and the intracellular spaces of densely packed neurons in the gray matter. From an electrochemical perspective, neurons can be interpreted as batteries, and SD resembles a slowly spreading chain reaction of short circuits in these batteries, releasing almost all of the electrochemical energy stored in them.^11,20^ It represents the most extreme measurable deviation from cellular homeostasis observed in the living CNS and is the only known event associated with a measurable increase in tissue temperature in preparations devoid of blood circulation such as brain slices.^22,23^ In other words, SD can result in a measurable increase in tissue temperature independently from perfusion changes. This results from the near-complete release of the electrochemical energy that is normally stored in neurons. The thermodynamic principles underlying this release of free energy are well understood and have been reviewed previously.^20^

Notably, the changes in ions and other small molecules, including neurotransmitters, during SD exceed those observed during an epileptic seizure—the second most extreme deviation from cellular homeostasis in the CNS—by several orders of magnitude.^6^ Clinicians should recognize that SD and epileptic seizures are fundamentally different. For example, a common mistake is classifying SD within a framework of excitation and inhibition, that is, commonly used in the context of epileptic seizures, but fails to capture the nature of SD. Contrary to seizures, both inhibitory and excitatory neurons show a near-complete depolarization during SD, lose their normal function, and release large quantities of their respective neurotransmitters.^24,25^ Also, while loss-of-function mutations result in epilepsy syndromes, SD can be triggered by gain-of-function mutations in genes such as the Na^+^ channel gene NaV1.1, which are mainly expressed by GABA-ergic interneurons.^26–29^ On the other hand, there are also conditions that increase the susceptibility to both SD and seizures.^30–33^

**Figure 1.**
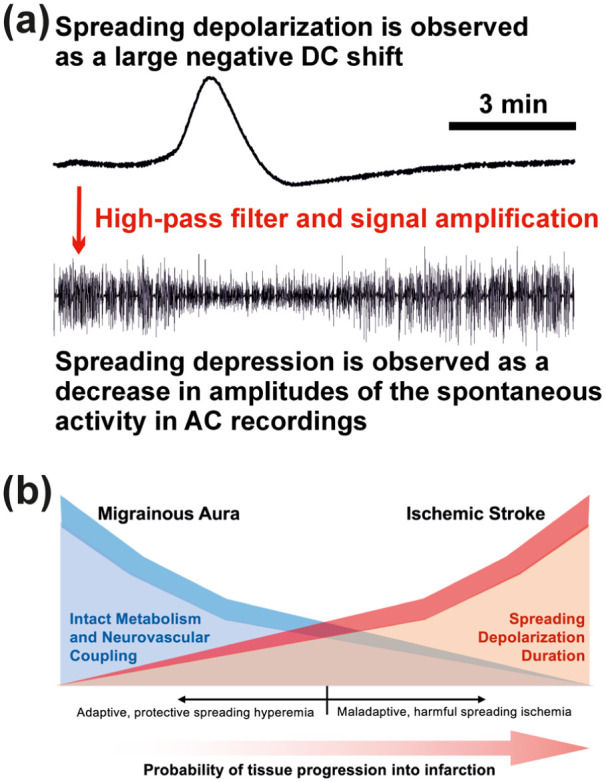
(a) Spreading depolarization and depression features in ECoG. There are numerous ways to measure SD. However, the most common is DC–ECoG. When SD occurs in nervous tissue with spontaneous activity, it usually leads to a spreading depression of spontaneous activity which is measured in the AC frequency range of the ECoG. This recording was performed using a subdural platinum-iridium electrode in a patient. It is noteworthy that SD can also occur in nervous tissue that is so severely metabolically impaired that it no longer exhibits spontaneous activity. Under these circumstances, SD can of course not induce spreading depression. The ECoG traces are oriented with negativity upward according to the EEG convention. (b) Illustrative representation of the stroke-migraine SD continuum. The term SD continuum describes the spectrum from transient events with negative DC shifts and neuronal cytotoxic edema of short to intermediate duration in adequately supplied or mildly ischemic tissue to terminal events in severely ischemic tissue characterized by long-lasting DC shifts, long-lasting cytotoxic edema and transition of the neurons from the state of injury to cell death.^1,6^ Importantly, the roles of different ion channels, transporters and membrane pumps in the SD process seem to change along the continuum from SD in normal tissue to SD in ischemic tissue. This is the basis for fundamental changes in pharmacosensitivity along the SD continuum, which unfortunately complicates the development of a treatment.^6^ The hemodynamic responses to SD also show a continuum across cerebral gray matter. At one site, SD may be accompanied by a vasodilatory response (spreading hyperemia),^53^ while at another site it may be accompanied by a vasoconstrictive response (spreading ischemia).^57^ Spreading ischemia can be the sole cause of widespread cortical infarcts.^58^ AC: alternating current; DC: direct current; ECoG: electrocorticography; SD: spreading depolarization.

**Figure 2.**
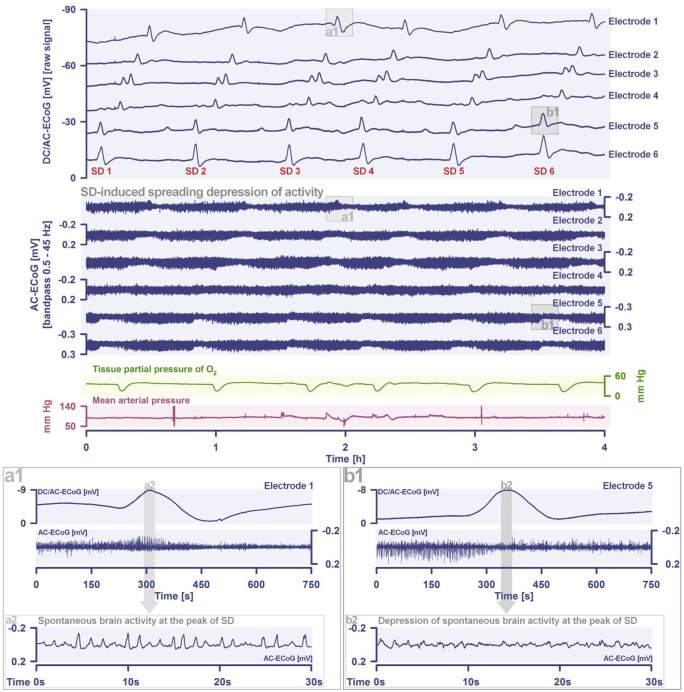
Cluster of SDs in a 55-year-old woman with SAH and intracerebral hematoma due to a ruptured aneurysm of the right middle cerebral artery. In addition, this patient from the DISCHARGE-1 trial^70^ developed an MRI-proven delayed ischemic infarct. In total, 191 SD were recorded in the patient during 12.1 days of continuous recording time. The recording shown is from day 3 after SAH. Traces 1–6 from top to bottom show the DC/AC–ECoG recordings (band-pass: 0–45 Hz) where SD are observed as large negative DC shifts. The ECoG traces are oriented with negativity upward according to the EEG convention. Traces 7–12 show the spontaneous activity in the AC frequency band between 0.5 and 45 Hz. Trace 13 displays changes of the tissue p_ti_O_2_ measured with an intraparenchymal sensor. Note that each SD leads to a drop in p_ti_O_2_. Trace 14 gives the mean arterial pressure measured with a catheter in the radial artery. At a higher temporal resolution, (a1) displays an SD with activity increase (boom)^37^ during the negative DC shift and (b1) with the more common activity depression. In the tracks below (a2, b2), the spontaneous activity during the respective SD is shown in even higher temporal resolution. The two SD shown in (a, b) are highlighted in the top panel by the gray boxes. AC: alternating current; DC: direct current; ECoG: electrocorticography; p_ti_O_2_: partial pressure of oxygen; SAH: subarachnoid hemorrhage; SD: spreading depolarization.

SD propagates through contiguous cerebral gray matter at a rate between 2 and 9 mm/min, while sparing white matter.^34–36^ The near-complete collapse of electrochemical gradients during SD, and resulting influx of cations into neurons, can be observed as a large negative direct current (DC) shift of the extracellular field potential. In patients, this is measured with subdural electocorticography (Figure 1(a)).^21^ In electrically active tissue, SD often leads to a spreading depression of activity, which is recorded as a decrease in amplitudes of spontaneous activity in alternating current (AC) electrocorticography. Even though the terms “spreading depolarization” and “spreading depression” have sometimes been used interchangeably, they have distinctive meanings: “spreading depression” refers to the functional suppression of cerebral activity, whereas “spreading depolarization” describes the sustained depolarization and near-complete disruption of the electrochemical transmembrane gradients associated with neuronal cytotoxic edema. Thus, spreading depression occurs as just one of the consequences of SD and generally cannot occur without SD as the underlying cause. SD, on the other hand, can occur without spreading depression, since SD waves can propagate through severely injured and functionally silent (isoelectric) tissue that, by definition, cannot be further depressed. In some cases, as when measured at the brain surface, SD can also cause an increase in spontaneous activity, the so-called “boom”, rather than a depression during the depolarization phase (Figure 2).^37^ Mechanistically, the boom results from volume conduction of signals from spared delta generators in deep cortical layers during an SD confined to superficial cortical layers as well as moderate neuronal depolarization and sustained excitation organized in gamma oscillations in a narrow sub-SD zone. Such genuine changes in spontaneous activity during SD must be distinguished from artifacts, such as bowtie-shaped pulse artifacts in isoelectric tissue, which arise from changes in tissue elasticity during SD.^32,38^

Although neurons passively enter the SD state, recovery from this state requires a large amount of energy, as membrane pumps such as Na^+^/K^+^-ATPases and Ca^2+^-ATPases must work beyond normal levels to restore electrochemical gradients associated with the normal physiological state.^6^ Therefore, even in otherwise normal tissue, SD increases the cerebral metabolic rate of oxygen^39,40^ and depletes neuronal and tissue ATP concentrations.^41–43^

It shall also be mentioned here that SD is definitely not a hibernation state. In contrast to neurons in the SD state, those in torpor, as seen in hibernating animals, maintain their resting membrane potential, input resistance, rheobase, and capacity to generate action potentials.^44^

## SD as an adaptive and maladaptive response in the course of evolution

SD is a highly conserved CNS response to various forms of acute neuronal and glial stressors. It is observed across different animal species, including mammals, all other properly investigated vertebrates, and various invertebrates.^45–49^ The origin of this relatively primitive, massive, slowly propagating reaction to injury events may even be traced back to the plant kingdom.^50,51^ Nonetheless, we are still far from establishing a complete phylogenetic classification of SD. Even within mammals, it has not yet been systematically investigated whether there are processes outside the CNS that are related to SD.^52^ However, it could also be that the SD phenomenon is CNS-specific. Speculatively, SD could be related to the specific shielding of the CNS from the rest of the body by the blood-brain barrier.

**Figure 3.**
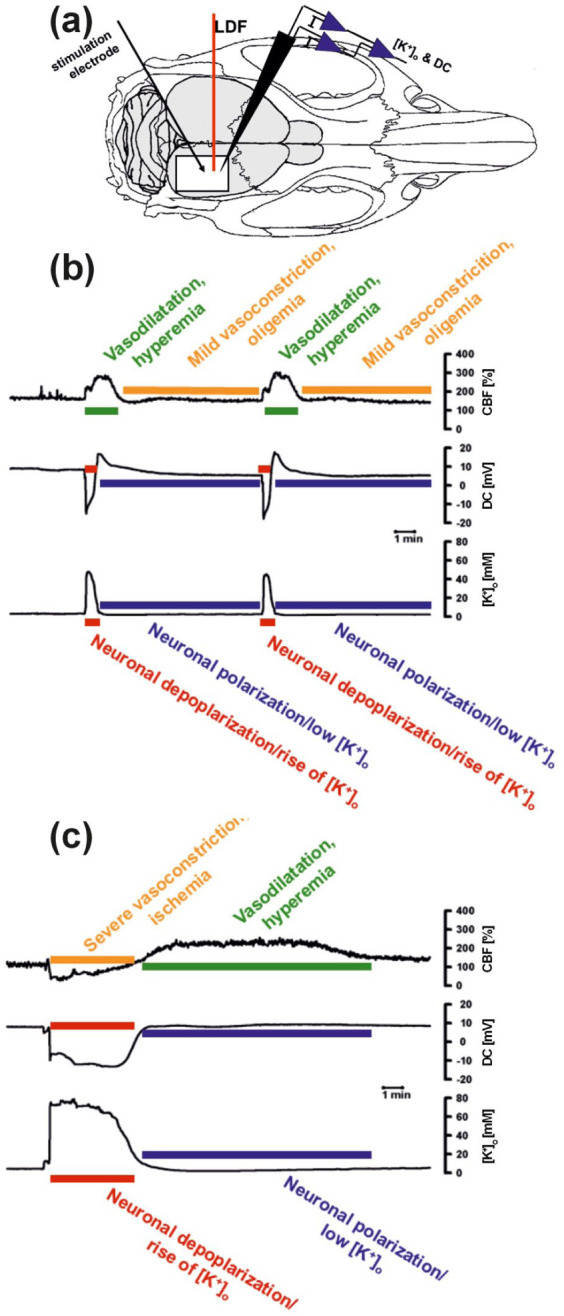
Illustration of the two opposing hemodynamic responses to SD experimentally induced with electrical stimulation in rats: (a) LDF captures real-time changes in rCBF (% from baseline), while the intracortical microelectrode simultaneously records DC potential and extracellular K^+^ concentration. (b) In the normal hemodynamic response, SD is characterized by a negative DC shift and an increase in extracellular K^+^, coupled with a transient increase in rCBF (spreading hyperemia), followed by mild oligemia after neuronal repolarization. (c) Conversely, in the inverse hemodynamic response, SD is characterized by a more sustained and prolonged depolarization accompanied by a transient decrease in rCBF (spreading ischemia), followed by hyperemia after repolarization—if the neurons survive. DC: direct current; LDF: laser-Doppler flowmetry; rCBF: regional cerebral blood flow; SD: spreading depolarization.

**Figure 4.**
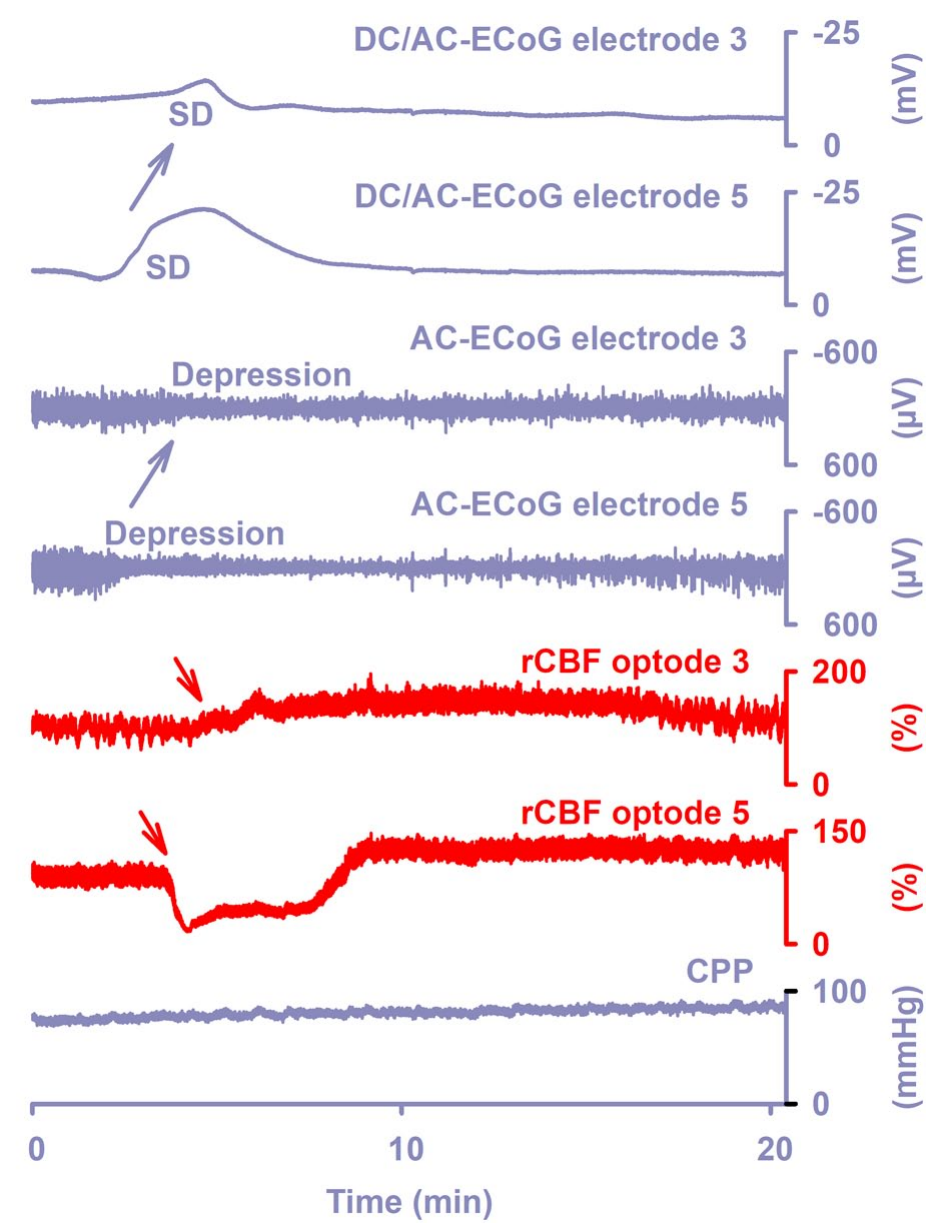
The upper two traces show the large negative DC shift that indicates SD (band-pass: 0–45 Hz). Traces 3 and 4 show the spreading depression in response to SD as a decrease in amplitudes of spontaneous activity in AC-ECoG (band-pass: 0.5–45 Hz). Traces 5 and 6 display the responses of rCBF to SD as measured with optodes adjacent to electrodes 3 and 5 using laser-Doppler flowmetry. Trace 7 shows CPP, which is calculated by subtracting the intracranial pressure measured via an external ventricular drain from the mean arterial pressure measured in one of the radial arteries. While SD caused spreading hyperemia at optode 3 (= normal hemodynamic response), it caused spreading ischemia at optode 5 (= inverse hemodynamic response; red arrows). Accordingly, negative DC shift and activity depression were short-lasting at electrode 3, while they were longer-lasting at electrode 5. Recovery from SD requires a large amount of energy, as membrane pumps such as Na^+^/K^+^-ATPases and Ca^2+^-ATPases must work beyond normal levels to restore electrochemical gradients associated with the normal physiological state. If rCBF does not increase during the depolarization phase of the SD but instead decreases significantly, this leads to delayed repolarization and delayed recovery of neuronal activity (traces 2 and 4 in comparison to traces 1 and 3). Electrodes 3 and 5 are 2 cm apart. Note the spread of SD and activity depression (blue arrows). The figure shows original recordings from a patient with subarachnoid hemorrhage.^60^ AC: alternating current; CPP: cerebral perfusion pressure; DC: direct current; ECoG: electrocorticography; rCBF: regional cerebral blood flow; SD: spreading depolarization.

A fundamental principle of SD is that its impact—ranging from harmful to benign or even potentially beneficial—depends on the specific (patho)physiological context. This effect can vary between neighboring brain regions, which is illustrated, for example, by differences in the hemodynamic response. In rats and phylogenetically younger species, SD acts as a potent stimulus to increase regional cerebral blood flow in otherwise normal tissue (= spreading hyperemia; Figure 3).^12,16,53^ With a delay after repolarization, spreading hyperemia is typically followed by physiological, sustained, moderate hypoperfusion (= oligemia).^54,55^ This “normal hemodynamic response” is adaptive and the tissue recovers fully from SD in this case.^56^ However, when the neurovascular unit is dysfunctional, SD can also lead to severe vasoconstriction rather than vasodilation during the neuronal and glial depolarization phase, resulting in a long-lasting, expanding local perfusion deficit called spreading ischemia (Figure 3).^11,57^ Spreading ischemia delays (i) the restoration of ionic gradients, (ii) neuronal and glial repolarization, and (iii) recovery from cytotoxic edema. In addition, it promotes extracellular ionic edema as it increases the glymphatic inflow speed of cerebrospinal fluid into the tissue.^5^ This “inverse hemodynamic response” is maladaptive. Even if cerebral perfusion was normal immediately before the onset of SD, the spreading ischemia caused by SD can be so severe and long-lasting that it ultimately leads to extensive infarcts.^58,59^ This full spectrum from normal to inverse hemodynamic responses has also been demonstrated in patients using subdural electrode strips with embedded laser Doppler probes (i.e. optoelectrodes) to measure cerebral blood flow in the same tissue.^11,35,59–61^ Illustrating the dependence of SD’s manifestations on local tissue conditions, the same SD wave can occur in one brain region with a normal hemodynamic response and yet provoke the inverse hemodynamic response in another region (Figure 4).^62^ Interestingly, mice show hemodynamic responses that deviate somewhat from those of phylogenetically higher species.^63–65^ Insects have SD just like vertebrates,^66^ but they have an open circulatory system of hemolymph instead of a closed blood circulation. To our knowledge, how their hemolymphatic system reacts to SD has not yet been investigated. What is certain, however, is that SD have existed phylogenetically longer than species with a closed arteriovenous blood circulation.

## SD in the clinical context: A universal response

**Figure 5.**
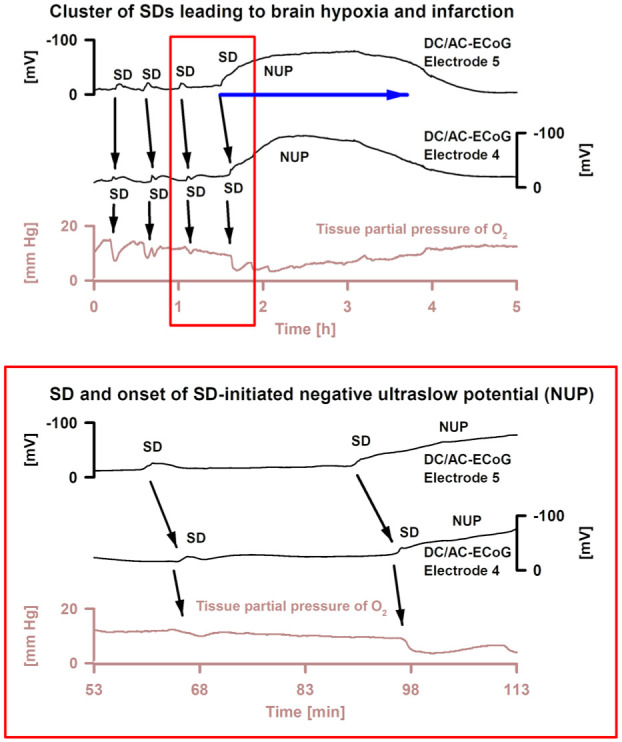
Serial neuroimaging-confirmed ECoG evolution towards delayed brain infarction in a 50-year-old woman with subarachnoid hemorrhage (original recording^59^). SD typically increases severity several hours before the ischemic events. Only the actual ischemic event (blue arrow) shows severe and prolonged SD-induced hypoxia and the SD-initiated NUP, leading to brain infarction. Note that drops of tissue pO_2_ (intraparenchymal sensor near electrode 4) follow SD in time as a result of inverse neurovascular coupling and increased cerebral metabolic rate of O_2_. The lower panel shows the red box of the upper panel with a higher temporal resolution. The two subdural electrodes are placed on the brain surface at a distance of 1 cm. Note the spread of SD (black arrows). AC: alternating current; DC: direct current; ECoG: electrocorticography; NUP: negative ultraslow potential; SD: spreading depolarization.

**Figure 6.**
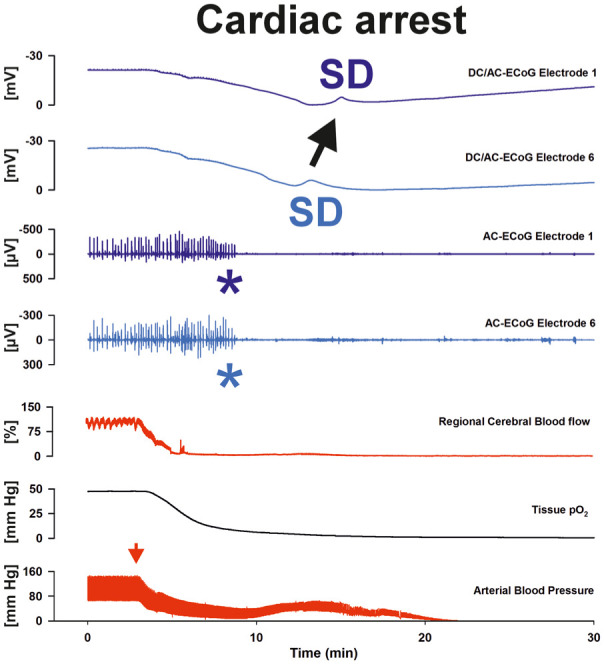
Recording of the terminal SD following cardiac arrest in a patient. In parallel with the fall in arterial blood pressure (red arrow, measured in the radial artery), laser-Doppler flowmetry (subdural optode) shows a rapid and sustained loss of cortical perfusion (regional cerebral blood flow), followed by severe cerebral hypoxia (tissue pO_2_). A few minutes later, subdural platinum–iridium electrodes capture first a non-spreading depression of spontaneous brain activity in electrodes 1 and 6 (asterisks in traces 3 and 4, AC–ECoG). This is followed by a large negative shift in the DC potential which propagates from electrode 6 to electrode 1 (traces 1 and 2), indicating the onset of terminal SD. This initially reversible wave of SD reflects the near-complete collapse of transmembrane ion gradients and precedes the loss of neuronal viability in the context of complete and prolonged energy failure (original recording^75^ in a 56-year-old woman). AC: alternating current; DC: direct current; ECoG: electrocorticography; SD: spreading depolarization.

SD has been unequivocally documented in humans as a critical reaction to a wide range of neurological conditions, encompassing cerebral ischemia,^67^ intraparenchymal hemorrhage,^68^ subarachnoid hemorrhage (SAH; Figure 5),^69–71^ chronic subdural hematoma,^72^ moyamoya vasculopathy,^73^ and TBI.^74^ It has also been documented in the process of brain death, whether occurring after cardiac arrest (Figure 6) or during continued systemic circulation.^70,75–78^ In neurocritical care, regional electrocorticographic monitoring of SD enables the detection of injury even in remote areas, as SD can propagate far from ischemic or metabolically stressed brain regions.^21^ Therefore, SD are an interesting biomarker for real-time identification of impending and ongoing brain injury, especially in comatose patients.^67,70,74^ SD has also been shown to alternate with epileptic seizures in patients with status epilepticus in the context of acute cerebral injuries.^31–33^ In addition, direct and indirect evidence in humans supports the occurrence of SD in the context of non-migrainous and migrainous aura.^12,13,54,79^ In line with this, genetic animal models have suggested that SD plays a critical role in familial hemiplegic migraine (FHM).^26,65,80,81^ Moreover, SD has been experimentally demonstrated in at least one entity of hereditary movement disorders.^82^ Increasing evidence also suggests that SD, besides epileptic seizures, could play a role in the progression of brain tumors.^83–85^ Brainstem SD could be the trigger for sudden unexpected death in epilepsy (SUDEP).^86,87^ SD could also be involved in various post-seizure phenomena, including postictal ambulation.^88^ Therefore, while susceptibility to developing SD may vary across different CNS tissues, pathological conditions, and among individuals, SD itself is arguably a universally conserved response.

## SD following global and focal cerebral ischemia

Perhaps the most revealing clue to the nature of SD is its occurrence in the brain’s gray matter following global cerebral ischemia (cardiac arrest) and focal cerebral ischemia. A common misconception about both of these conditions is that they lead to a gradual loss of ATP in the brain’s gray matter until the neuronal ATP reserves are more or less fully depleted. This would occur within 5–10 min and then culminate in the immediate death of the affected neurons. In the case of focal cerebral ischemia, SD (often referred to as peri-infarct depolarizations) would then emerge from the necrotic core and invade the ischemic penumbra. However, this understanding is incorrect and leads to misconceptions about the mechanisms of cell and brain death, the timing of their development, opportunities for intervention, and the role of SD.

**Figure 7.**
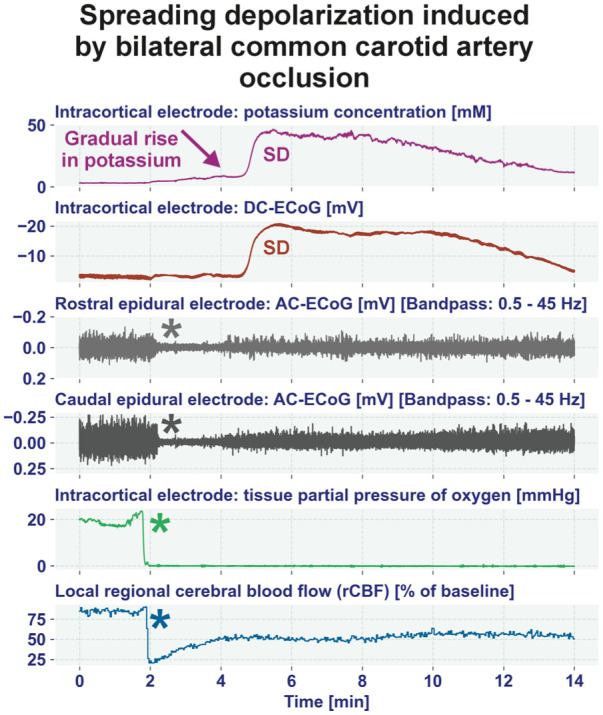
Example of an original recording during the initial period of BCCAO in a spontaneously hypertensive stroke-prone rat. The occlusion induced (i) a steep and rapid fall of rCBF (blue asterisk), which partially recovered shortly thereafter (trace 6 from top to bottom), (ii) a drop of tissue partial pressure of oxygen (trace 5) (green asterisk), and (iii) a non-spreading depression of spontaneous brain activity (gray asterisks in traces 3 and 4). With a delay of more than 2 min after BCCAO, SD began, observed as a negative DC shift (traces 2) and as a marked increase in the [K^+^]_o_ (trace 1). Note the gradual increase of [K^+^]_o_ before the SD which distinguishes SD in ischemic tissue from SD in normal, metabolically intact tissue (compare Figure 8).^6^ This gradual rise in [K^+^]_o_ before the SD presumably results from activation of neuronal ATP-sensitive and G protein-dependent Ca^2+^-sensitive K^+^ channels and impaired Na^+^/K^+^-ATPase function.^212–214^ The ECoG traces are oriented according to the convention of EEG with negativity up and positivity down. Note that Clark-type polarographic O_2_ microelectrodes such as the OX-10 by Unisense A/S (Aarhus, Denmark) consume oxygen for the measurement and are therefore no longer able to measure fluctuations in oxygen tension in the low oxygen tension range. BCCAO: bilateral common carotid artery occlusion; DC: direct current; ECoG: electrocorticography; EEG: electroencephalography; [K^+^]_o_: extracellular potassium concentration; rCBF: regional cerebral blood flow.

In contrast to this scenario, numerous studies have conclusively shown that an SD wave emerges in tissue at 1–5 min after the onset of severe ischemia, whether in cardiac arrest or focal ischemia (for a selection of studies describing this phenomenon in different species and conditions see Table 2). The decrease in intraneuronal ATP is relatively small initially, and only drops steeply after the onset of this SD,^43^ and the same is true for changes in intra- and extracellular ion concentrations (Figure 7). Importantly, the neurons remain alive through this sequence and only begin to die following the onset of SD. If the energy supply is restored in time, the SD can be reversed (i.e. the tissue will repolarize) and the neurons may survive. If the energy supply is not restored, the depolarized state will persist and neurons will die.^59^

**Table 2.** Evidence of “spreading” depolarization prior to neuronal death in the ischemic core of focal cerebral ischemia and prior to whole brain death.

References	Setting	Species	Age/weight	Sex	Method	Condition	Original recording
Leão^8^	In vivo	Rabbit	–	–	DC potential	Three-vessel occlusion (BCCAO + basilar artery occlusion)	Original description of the spread
Dijkhuizen et al.^100^	In vivo	Rat	300–400 g	Male	DC potential	MCAO	Figure 2
Jarvis et al.^215^	In vitro, cortex slices	Rat	21–30 days	Male	DC potential, IOS	Oxygen–glucose deprivation	Figures 1 and 2
Murphy et al.^206^	In vivo	Mouse	2–5 months	Mainly males	Two-photon microscopy, IOS, electro-physiology	BCCAO	Figure 9
Oliveira-Ferreira et al.^167^	In vivo	Rat	250–380 g	Male	DC potential	Brain topical endothelin-1 application	Figures 1 and 4
Oliveira-Ferreira et al.^167^	In vivo	Human	44–71 years	Both	DC potential	SAH, expansion of intracerebral hematoma	Figure 5
Farkas et al.^216^	In vivo	Rat	500–560 g	Male	Voltage-sensitive dye	Circulatory arrest	Figure 2
Drenckhahn et al.^97^	In vivo	Human	49–68 years	Both	DC potential	SAH, delayed brain infarction	Figure 5
Hartings et al.^49^	In vivo	Swine	32–40 kg	Female	DC potential	Sulcal clot model of SAH	Figure 4
Hartings et al.^49^	In vivo	Human	22–78 years	Both	DC potential	SAH, early brain infarction	Figure 7
Lückl et al.^59^	In vivo	Human	33–68 years	Both	DC potential	SAH, delayed brain infarction	Figures 3(c), 4, and 6
Dreier et al.^75^	In vivo	Human	29–78 years	Both	DC potential	TBI and SAH, circulatory arrest	Figures 1 and 3–5
Carlson et al.^76^	In vivo	Human	57 years	Female	DC potential	SAH, brain death during continued systemic circulation	Figures 2–4
Dreier et al.^77^	In vivo	Rat	10–16 weeks	Male	DC potential	Hypoxia, circulatory arrest	Figures 1–4
Dreier et al.^77^	In vivo	Human	40–85 years	Both	DC potential	SAH, brain death during continued systemic circulation	Figures 7 and 8
Mestre et al.^5^	In vivo	Mouse	8 weeks	Male	GCaMP fluorescence	MCAO	Movie 5
Robertson and van Dusen^66^	In vivo	*Locusta migratoria*	3–6 weeks	Both	DC potential	Anoxia	Figure 8
Zhao et al.^217^	In vivo	Mouse	4 months–1 year	Female	GCaMP fluorescence	Photothrombosis	Video S1
Vinokurova et al.^218^	In vivo	Human	64 years	Female	DC potential	SAH, delayed brain infarction	Figure 7
Dreier et al.^70^	In vivo	Human	22–82 years	Both	DC potential	SAH, delayed brain infarction, brain death during continued systemic circulation, circulatory arrest	Figures 5 and 8
Dreier et al.^78^	In vivo	Human	26–70 years	Both	DC potential	SAH and TBI, brain death during continued systemic circulation	Figures 5–7
Sword et al.^48^	In vivo	Monkey	23–32 years	Both	DC potential	MCAO and subsequent circulatory arrest	Figures 3 and 5–7

BCCAO: bilateral common carotid artery occlusion; DC: direct current; GCaMP: genetically encoded calcium indicator; IOS: intrinsic optical signal; MCAO: middle cerebral artery occlusion; SAH: subarachnoid hemorrhage; TBI: traumatic brain injury.

The table provides an overview of studies in which it has been investigated that an orderly spreading, initially reversible SD wave starts from one point in the tissue before the neurons die. The spread of SD was documented in figures and videos of original recordings. The studies were performed in different species and different conditions of severe energy deficiency states.

**Figure 8.**
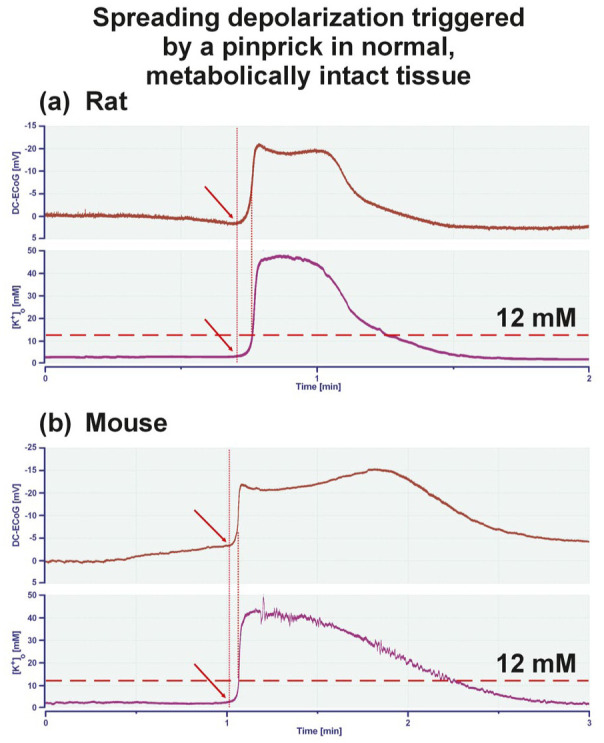
(a) Using a K^+^-sensitive microelectrode, an SD was recorded in normal, metabolically intact neocortex in vivo in a Wistar rat under thiopental anesthesia. (b) Recording of an SD in normal, metabolically intact neocortex in vivo in a C57BL6 mouse under urethane/α-chloralose anesthesia. Both SD were triggered by a pinprick several millimeters away from the recording location. The negative DC shift in ECoG (upper curve) and the increase in [K^+^]_o_ (lower curve) occurred virtually simultaneously. If at all, the negative DC shift preceded the rise in [K^+^]_o_ consistent with Somjen’s statement that “*the extracellular potassium concentration does not start to increase ahead of the negative DC shift*”^19^ (authors’ emphasis). Arrows mark the onset of SD. DC: direct current; ECoG: electrocorticography; [K^+^]_o_: extracellular potassium concentration; SD: spreading depolarization.

Based on these observations, SD could be described as an orderly retreat from life, a retreat program that can be activated as if with a switch. This orderly retreat seems to be the usual way in which the gray matter of the CNS undergoes acute death. Therefore, perhaps the most important fact about SD is that it describes the usual dying process of the brain’s gray matter. However, it is important to note that, as long as the tissue is in the process of dying, it is not yet dead and therefore can either transition into dead tissue or survive. In other words, in the gray matter of the CNS, SD is usually the last potentially reversible tissue state before death. This goes hand in hand with the interpretation of SD not only as an orderly retreat from life that precedes dying, but also as a potential reboot. When cell death occurs, the onset of SD marks the countdown to cell death, but not yet cell death itself.^6,78,89,90^ Neurons only begin to die when SD persists beyond a certain duration, known as the “commitment point.”^91^ If reperfusion occurs and neurons repolarize before this commitment point, they can survive.^59,89^ For example, following occlusion of the middle cerebral artery (MCAO) in rats, neuronal death begins only after SD has persisted for more than 15 min.^59,78^ If reperfusion occurs within this time window, no neuronal death is observed in histological specimens 72 h later. Similarly, SD is also reversible in the initial stages following cardiocirculatory arrest if perfusion is restored in time.^92,93^

The timing of the commitment point depends on multiple factors,^20,94^ with the degree of residual tissue perfusion being the most critical.^95,96^ After the commitment point, the affected neurons will die regardless of whether reperfusion is achieved. However, the timing of reperfusion after the commitment point may still have an influence on the mode of cell death (e.g. apoptosis vs necrosis).^78^ From an electrophysiological standpoint, the depolarization state beyond the commitment point is no longer referred to as SD but as the negative ultraslow potential (NUP).^11,49,97^ The so-called terminal SD thus consists of an initial, still reversible SD component that progressively transitions into the NUP (Figure 5). Unfortunately, it is currently not possible to determine the exact onset of the NUP based on electrocorticographic recordings. Thus, the distinction between the initial reversible SD component and NUP is largely theoretical. Practically, the longer the local duration of SD, the greater the likelihood that subsequent neuroimaging or histology will reveal a new infarct in the electrode’s recording area.^21,59^

In focal cerebral ischemia, after the occurrence of the first SD, subsequent further SD can occur in the following hours to days due to the persistent energy supply-demand mismatch in the ischemic penumbra.^67,98^ These recurrent SD can exacerbate neuronal injury through prolonged ionic breakdown, contributing to the progressive infarct growth over many hours,^99–102^ even though not every SD in the vicinity of an ischemic zone necessarily leads to an increase in histological damage,^103^ as this depends on the local duration of the individual SD.^78^ Overall, it can be assumed that patients who have suffered an infarct involving the gray matter of the brain usually had one or more SD, particularly before cell death occurs in the tissue that later appears as an infarct on MRI. Indeed, SD are accompanied by specific MRI changes, such as hyperintensity on diffusion-weighted imaging and reduced apparent diffusion coefficient values.^6,104–106^ These changes arise from SD-induced beading of neuronal dendrites, which impairs diffusion of water along the dendritic main axes. Initially, both the MRI changes and the SD themselves are reversible.^104,107^ However, both become irreversible if SD evolves locally into a NUP (i.e. tissue death). It should be noted that SD never develops from necrotic tissue, but always requires viable tissue to form and spread. In the course of SD, the tissue either dies or it recovers from the SD and survives. Not only early but also delayed SD can manifest clinically as a new transient or permanent neurological deficit.^6,69,70^ It is important to acknowledge that there are exceptions to these general rules. For example, it has been previously shown that selective ischemic death of Purkinje cells can evolve without the occurrence of SD.^38^

After focal cerebral ischemia, it is estimated that the final size of the lesion in diffusion-weighted imaging is only reached after ~70 h in patients.^108^ In agreement with this, Kowoll et al. found that in ~50% of patients with malignant hemispheric stroke (MHS) who underwent an initial MRI scan 57 ± 41 h after the onset of MCAO, a second MRI scan after 139 ± 28 h still showed infarct progression of >5%.^67^ Following permanent MCAO in non-human primates, the full lesion volume was also only reached later than 48 h after the onset of ischemia,^109^ whereas in rodents this was the case within 3–6 h.^110^ To put it simply: 60 min of ischemia in a primate corresponds to a significantly shorter period of ischemia in a rodent in terms of the extent of damage caused. The reason why cell damage progresses faster in rodents than in primates is probably because rodents generally have a significantly faster metabolism than primates.^111^ Reperfusion modifies the temporal course. After an initial recovery phase, delayed cell death typically occurs, which in rodents is assumed to take place between 6 and 12 h in the area of the primary energy failure.^112^ Overall, infarct growth in rodents may continue for up to 24 h after the onset of transient MCAO.^113^

## Migrainous infarction

At the forefront of the SD wave in normal tissue, nearby neurons in otherwise healthy tissue often exhibit synchronized initial firing, known as epileptoid activity.^114^ This activity locally lasts 1–5 s and presents as a high-frequency burst of population spikes, followed by a depolarization block (i.e. the SD) that suppresses action potential generation. The electrocorticogram reflects this suppression as a spreading depression of spontaneous activity. Notably, the depression phase outlasts the depolarization phase, indicating that other mechanisms beyond the depolarization block are involved in the depression, such as accumulations of (i) extracellular adenosine and (ii) intracellular Zn^2+^ and Ca^2+^.^115–117^ These changes in spontaneous activity translate into the acute presentation of clinical symptoms experienced by migrainous aura patients: an initial phase of brief, spreading, positive symptoms (e.g. flickering in a visual field or tingling of a body part), corresponding to the epileptoid activity, is followed by a phase of negative symptoms (e.g. a visual scotoma or numbness of a body part) corresponding to activity depression.^6,16,118^ The symptoms show a characteristic slow spread across different modalities such as vision or somatosensation, without, usually, resulting in lasting damage.

Although patients with ischemic stroke usually experience more frequent and longer SD than patients with migrainous aura,^119^ they typically present with a sudden, large-scale, multimodal neurological deficit, rather than the slowly propagating symptoms of migrainous aura. The electrophysiological correlate of the sudden, large-scale, multimodal neurological deficit typical of cerebral ischemia is not the SD-induced spreading activity depression (Figure 1(a)), but the so-called non-spreading activity depression (Figure 7).^6,120^ Non-spreading depression is characterized by hyperpolarization of neurons^121^ and occurs in ischemic tissue before the onset of the first SD. It was first described by Leão in 1947 and usually develops within 20–40 s in areas with a cerebral blood flow below a critical threshold of ~22 ml/100 g/min.^11^ Notably, SD cannot trigger spreading depression in isoelectric tissue following non-spreading depression as there is no activity that could be depressed.^21^ However, in cases of focal ischemia, SD can invade tissue further from the severe perfusion deficit where non-spreading depression is less pronounced, leading to the occurrence of spreading depression.

It is, therefore, conceivable that a punctiform, localized ischemia can trigger SD and spreading depression that travels through normal eloquent brain tissue, resulting in an aura phenomenon. Olesen et al.^122^ argued that such ischemia-induced non-migrainous auras are more common than migrainous aura-induced ischemic insults, aka migrainous infarcts. For example, ischemia-induced auras can represent the initial symptom of carotid artery stenosis.^122,123^ Considering that SD are abundantly measured in the tissue surrounding human brain infarcts,^119^ it is not surprising that one of these SD can occasionally trigger an aura, although this occurs relatively rarely. This rarity could stem from the short distances that SD typically travel in human brains from the ischemic zone to metabolically intact tissue, as evidenced by intraoperative laser speckle contrast analysis imaging in patients with MHS.^35^ Similarly, auras associated with a patent foramen ovale or a reversible cerebral vasoconstriction syndrome likely result from ischemia-induced SD.^89,90,124–126^ Notably, these scenarios do not by definition constitute a migrainous infarction simply because an aura occurs alongside a cerebral infarction.

Following the International Classification of Headache Disorders-3 (ICHD-3), migrainous infarction is defined as the presence of one or more typical aura symptoms associated with clear imaging evidence of ischemic infarction in an individual with an established history of migraine with aura. The decisive factor is that this infarct must be localized precisely in the area of the brain that correlates with the reported aura symptoms. A little historical context may help to understand this definition. Historically, migrainous infarcts were considered evidence for the vascular theory of migraine, which stated that the migrainous aura is caused by cerebral vasospasm leading to ischemia, while the headache is caused by extracranial vasodilation.^14,127^ Leão’s SD was dismissed as “neuromythology” by proponents of this theory.^14^ However, 1981 marked a pivotal shift in migraine research, with Leão’s SD^9^ now being widely recognized as the underlying mechanism of migrainous aura. That year, studies measuring cerebral blood flow following intracarotid ^133^Xe administration provided evidence for the first time that migraine with aura attacks begin with focal hyperemia, followed by spreading oligemia and impaired functional activation—responses consistent with SD.^54,128^ Lauritzen emphasized in his 1994 review that oligemia during migrainous aura does not equate to ischemia.^16^ Later, Hadjikhani et al. confirmed and expanded these findings using functional MRI during migrainous aura.^12^

A modern interpretation of migrainous infarction emerged in 1998 with the discovery of the inverse hemodynamic response to SD (Figure 3).^57^ As explained previously,^11^ this response can develop in adequately supplied tissue where SD is not preceded by non-spreading depression. Therefore, SD can simultaneously trigger spreading depression (aura phenomenon) and severe microcirculatory vasospasm (cerebral infarction). However, just as SD and spreading depression are not exclusive to migraine, spreading ischemia is also not specific to migrainous infarcts. Studies using subdural optoelectrode strips and serial MRIs have demonstrated spreading ischemia in patients with SAH who developed delayed ischemic infarcts.^59,60^ Additionally, spreading ischemia has been recorded in the penumbra of MHS and following TBI in patients.^35,61^ In contrast, the hypothesis that stroke-like episodes in patients with mitochondrial encephalopathy with lactic acidosis and stroke-like episodes (MELAS) are related to spreading ischemia remains unverified by direct measurements and is based only on plausibility.^57^

Migraine with aura and ischemic stroke share SD as a pathophysiologic mechanism, which explains the numerous studies, including several meta-analyses, confirming the epidemiologic association between the two conditions.^129^ However, the relationship is likely complex and multi-faceted.^6^ Despite the overlap, it is unlikely that ischemia is the typical trigger of SD in patients with migraine with aura. If it were, we might expect an even stronger association between migraine with aura and ischemic stroke than is observed. Consistent with this, there are countless potential triggers of SD that have been demonstrated experimentally; ischemia is just one of them.^89,90^ Another unanswered question is why ischemic stroke, which is usually associated with longer-lasting and more frequent SD than migraine with aura, does not normally involve headache, if SD is the trigger for headache in migraine.^6^

## To treat or not to treat

The key question regarding any pathophysiological process is whether it is benign or harmful to health. If it is benign, it may be neglected; if harmful, it should be prevented. This raises the question of whether SD, a key component in the dying process of the brain’s gray matter, is ultimately good for health. While this question may sound odd, SD may actually provide neurons a chance to survive rather than leading to immediate cell death.^130^ The evolutionary conservation of SD could possibly be used as an argument for its potential health benefits. However, death itself is also a highly conserved evolutionary process and is obviously not conducive to health. Darwin’s theory of evolution states that traits are passed down from generation to generation if they represent an evolutionary advantage for the survival of the species rather than the individual.^131^ Even the death of individuals can be advantageous for the survival of the species when those have fulfilled their reproductive role and no longer compete for resources with those capable of reproduction. Evolutionary biologists have identified interesting examples of this.^132^ Evolution often follows counter-intuitive paths, requiring one to think a little “outside the box” to appreciate them.

A key aspect to be considered when interpreting the high conservation of SD in evolution may lie in the balance it strikes: traits like denser packing of neurons and enhanced synaptic transmission optimize CNS performance and may confer evolutionary advantages to the healthy individual at reproductive age, but may also increase the risk of network instability.^133^ This instability could pose the risk of SD as an accident in pathological conditions, the danger of which is usually high at the very beginning of life, then decreases during reproductive years before increasing again with advanced age. Notably, SD were reported not to occur in neonatal brains,^134,135^ which Somjen hypothesized protects the brain against the consequences of hypoxia, the risk of which is highest during birth.^91^ Rats then show the highest susceptibility to SD at a young age and then decreased susceptibility again late in life.^136,137^ The balance between network performance and stability may also be reflected in individual molecular components. For example, activation of N-methyl-D-aspartate receptors (NMDAR) is involved in the SD mechanism^138–142^ yet, on the other hand, is a key mechanism for the structural changes that occur in neurons during learning and memory formation.^143,144^ While low doses of the NMDAR antagonist MK-801 do not inhibit SD in the ischemic penumbra,^145,146^ high doses effectively prevent SD in mildly to moderately compromised tissue and provide significant neuroprotection in animal models of focal cerebral ischemia.^142,145–153^ Yet such doses impair both short- and long-term retention memory, illustrating the trade-off between physiological benefits and pathological risks.^154^ NMDAR may thus exemplify traits that enhance learning and memory in healthy individuals, outweighing the potential evolutionary downside of SD under pathological conditions. In the acute stage of neuronal injury, NMDAR antagonists may protect neurons, but may also hinder the compensation of neurological deficits through learning processes.^155^ Overall, experiments suggest that all known SD actors perform physiological functions in neuronal/astrocytic information processing. Their evolutionary advantage may therefore primarily reflect these physiological functions and only secondarily their significance for SD. In a simple scenario, evolution would have enabled neurons and astrocytes to optimize and increase their information processing power until the risk of SD, as an event comparable to a power grid collapse, outweighs the benefits. As a caveat, recent experimental findings on hippocampal SD contralateral to stroke suggest even greater complexity, as such SD had a beneficial effect on the recovery of physiological functions, namely contextual fear conditioning.^155^ However, this caveat is in turn limited by the well-founded hypothesis that transient global amnesia, which is associated with temporary loss of hippocampal function rather than improvement, is attributable to SD.^156^ So there are still many unanswered questions.

When considering whether SD are friend or foe, the results of the three large clinical studies on SD may provide further clues. In a prospective, observational, multicenter cohort study of 138 patients with TBI, SD clusters were independently associated with poor outcome.^74^ Similarly, in DISCHARGE-1, a prospective, observational, multicenter, cohort, diagnostic phase III trial involving 180 patients with SAH, SD burden (in particular, cumulative SD-induced depression durations per day) was independently predictive of early, delayed, and total brain damage, as well as mortality and outcomes at 7 months.^70^ Specifically, compared to patients without SD, those with at least one SD showed (i) a 3.1-fold higher relative risk and 42% higher absolute risk of poor outcomes, (ii) a larger volume of focal brain damage, and (iii) more abnormal brain tissue on longitudinal MRIs. SD were associated not only with delayed brain infarcts in this trial but also with reversible delayed neurological deficits (aka transient ischemic attacks).^70^ In a separate study involving 62 patients with MHS, the cumulative SD-induced depression duration per day was significantly longer in patients with infarct progression during the monitoring period, similar to the findings in SAH.^67^ Experimentally, the positive result of the meta-analysis of the above-mentioned animal studies with MK-801 strengthens the argument that SD are causally involved in damage progression during cerebral ischemia.^152^ Further supporting this causal connection, genetic predisposition to SD, for example, in mice carrying FHM1-Cav2.1 mutations, showed a remarkably increased vulnerability to larger infarcts when exposed to MCAO.^157^ In addition, even in the presence of normal supply of oxygen and glucose, neurons die within 1–2 h when exposed to prolonged SD triggered by an increase in the extracellular K^+^ concentration, persistent opening of Na^+^ channels, or inhibition of the Na^+^/K^+^ pump.^20^ Another potentially unfavorable property of SD is that it is a potent stimulus for neuroinflammation, as recently reviewed in the context of SAH.^64^

On the other hand, there are also convincing reports suggesting that short-lasting SD could have beneficial effects.^11,158^ In the healthy tissue surrounding the ischemic zone, SD upregulates growth factors and stress response proteins^49,159–162^ and may promote preconditioning,^163^ plasticity,^155,164^ and regeneration.^165,166^ Therefore, short-lasting SD could act as an alarm and defense signal to the tissue surrounding the area directly exposed to the stressor. Furthermore, SD-induced hyperemia may antagonize tissue acidosis in mildly ischemic tissue,^167^ while the physiological oligemia that follows SD in the surrounding adequately perfused tissue could mitigate the “steal phenomenon” on the already compromised perfusion within the ischemic zone.^167^ In the case of intracerebral hemorrhage, spreading oligemia may reduce the hematoma growth.^168^ Based on animal studies and preliminary clinical data without direct electrocorticographic evidence, it has also recently been suggested that seizure-induced SD may be the mechanism behind the antidepressant effect of electroconvulsive therapy.^169^ This is a very interesting hypothesis, although it could be another rather complex issue. For example, stroke increases the risk of major depression rather than reducing it,^170^ even though SD typically occur during its course.^67^ It also seems paradoxical in this context that migraine with aura and major depressive disorder show a high rate of comorbidity.^171^ Another potentially beneficial effect of SD proposed recently is a short-term intrinsic antiseizure effect.^172^ However, in patients with acute brain injury, this effect appears limited. While electrographic seizures are much less frequent than SD, they often follow in the wake of SD rather than precede and abort them.^31–33,133^ Another recent study found a strong positive association between SD and mortality, but neither a positive nor a negative association between SD and late epilepsy after SAH.^173^

Overall, neuromonitoring-guided multicenter trials will be needed to investigate whether interventions directly targeting SD can improve patient outcome. Tiered interventional feasibility studies such as INDICT and KETA-BID, which involve the NMDAR antagonist ketamine, are currently underway.^174,175^ They are based on several studies in patients with SAH, TBI, and MHS in which ketamine exhibited a suppressive effect on SD.^176–180^ While the unselective use of NMDAR antagonists at low doses in broad patient populations with stroke and TBI showed neither significant benefit nor harm,^181,182^ a precision medicine approach to testing NMDAR antagonists such as ketamine may yield different results. In these trials, for instance, the neuromonitoring of SD can be used to select patients most likely to benefit, and also to personalize therapeutic dosing and timing. Based on experimental studies, the NMDAR antagonist memantine is another interesting candidate alongside ketamine, as it has a less sedative effect than ketamine but also inhibits SD and led to improved neurological outcomes in a rat model of TBI.^183,184^ A completely different pharmacological goal would be to convert the inverse hemodynamic response that follows SD back into a normal hemodynamic response.^57,62,71^ This would turn harmful SD into harmless SD, without losing their potentially positive effects on metabolically intact tissue. Experimentally, nitric oxide (NO) donors and L-type Ca^2+^ antagonists such as nimodipine, for example, mitigate spreading ischemia.^57,62^ This is interesting, for example, in view of the occurrence of spreading ischemia in SAH patients who develop delayed ischemic infarcts,^59,60^ as a meta-analysis found that oral nimodipine prevented one-third of poor outcomes due to delayed cerebral ischemia, although it had no effect on angiographic vasospasm.^185^ In another clinical SAH study, cilostazol, which induces NO production by activating endothelial NOS, reduced the total duration of SD-induced depression per recording day and the occurrence of isoelectric SD consistent with a mitigating effect on spreading ischemia.^71^ SD as a potential pharmacological target in migraine has recently been reviewed elsewhere.^15^

## The reaction–diffusion mechanism of SD as a potential target of treatment: K^+^

When it comes to inhibiting SD and developing appropriate drugs, the focus is naturally on the mechanisms involved in the emergence and propagation of SD, which we will therefore discuss briefly. SD is generally considered to follow a reaction/diffusion mechanism.^186^ This means that neurons would release neuroactive substances that diffuse to neighboring neurons, where they trigger a self-propagating regenerative process. An argument for this hypothesis is, for example, that the fluid in which an isolated chicken retina was bathed during SD was able to trigger SD in another, otherwise untreated retina.^187^ The concept of the reaction/diffusion mechanism entails the re-induction of SD at every point in the tissue reached by the SD wave and might therefore well explain why SD propagation speed and susceptibility usually correlate.^34^ The two most frequently discussed molecules that have been implicated in the reaction/diffusion mechanism are K^+^ and glutamate. Although both molecules are undoubtedly involved in the SD process, it is controversial whether they are sufficient to explain it or whether essential pieces of the puzzle are still missing.^2,19,188–190^

For example, Somjen argued with regard to K^+^: “*Yet another problem is that, at a given point in the tissue, the extracellular potassium concentration does not start to increase ahead of the negative DC shift, as it should, if potassium were the agent of the propagation of SD*” (authors’ emphasis).^19^ This statement is controversial (e.g. it is challenged by the schematic drawing in Figure 1(b) in Pietrobon and Moskowitz^2^), yet is supported by the original recordings of SD (Figure 8), as triggered by a pinprick in vivo in a male rat and a male mouse. The figure shows that the extracellular potassium concentration ([K^+^]_o_) in a normal, metabolically intact cerebral cortex does not rise before the DC potential begins to swing toward negativity, but that both changes initially proceed almost exactly in parallel.

Hansen and Zeuthen (Figure 2 in Hansen and Zeuthen^191^) and Lehmenkuhler (Figure 1 in Lehmenkuhler^192^) reconstructed mean value curves of the DC potential and extracellular ion concentration changes from recordings of SD in the neocortex of naïve rats in vivo after triggering by a pinprick or remote K^+^ application. In our opinion, the shift of the DC potential toward negativity and the increase in [K^+^]_o_ began more or less simultaneously in the wavefront of SD in their figures, similar to Figure 8. However, it should be noted that it is subjective to a certain extent where exactly the viewer perceives the changes to begin. Very shortly after the onset of the negative DC shift and rise in [K^+^]_o_, pH showed an alkaline shift. About 4 s after the onset, extracellular pH and [Ca^2+^]_o_ started to fall. A moment later, [Na^+^]_o_ and [Cl^−^]_o_ dropped.

When performing such measurements, several aspects should be considered in order to avoid being misled. For example, to ensure that the DC potential and [K^+^]_o_ are measured in virtually the same sample volume, it is advisable to use a double-barreled electrode as used for the recordings in Figure 8, rather than two spatially separated electrodes for measuring the DC potential and [K^+^]_o_, as used, for example, in Enger et al.^193^ As SD propagates, its wavefront may reach two separate electrode tips at different times, which can lead to misinterpretations. Another aspect is that SD should not be triggered by K^+^ application if the aim is to investigate the temporal relationship between DC potential and [K^+^]_o_ in the wavefront of SD. A simple pinprick some distance away from the recording site, is more suitable for this purpose. After topical K^+^ application, the potential changes that occur at a distance before arrival of the SD wave have been shown, for example, by Kraig and Nicholson in the catfish cerebellum (Figures 4 and 5 in Kraig and Nicholson^194^). A peculiar DC positivity precedes the negative DC shift of SD in these experiments. Of note, Kraig and Nicholson did not count this DC positivity as part of the SD process. Presumably, it represents a current source due to glial buffering of K^+^ as it immediately followed pressure microinjection of 1 M KCl into the tissue. At the intersection of the glia-generated DC positivity preceding SD and the neuron-generated DC negativity during SD, it is in fact difficult to decide when the glial potential component ends and the SD starts. Accordingly, Kraig and Nicholson did not comment on the temporal relationship between negative DC shift and [K^+^]_o_ in their figure legend, but rather explained that the rise in [K^+^]_o_ preceded the fall in [Ca^2+^]_o_ during SD using the DC potentials of the two double-barreled ionsensitive microelectrodes as a reference point. In vivo in rats, it can be demonstrated that even at a distance of several millimeters from the K^+^ application site, there can be significant changes in spontaneous brain activity, which begin almost simultaneously with K^+^ application, long before the SD reaches the corresponding electrodes (see Figure 3 in Lemale et al.^20^). This means that the neural network can be significantly altered within a large radius around the local K^+^ application long before the SD reaches the outer perimeter of this circle. The size of the circle naturally depends on the application speed, the location and volume, and the concentration of the KCl solution used for SD induction, which is typically in the range of 1 or 2 M for puff application.

The following plausibility check, which takes into account the K^+^ threshold for SD, further challenges the hypothesis that the increase in [K^+^]_o_ is necessarily the initiator of SD in the propagation process: In order to trigger SD by artificially increasing [K^+^]_o_ in neocortex slices from normal 6- to 8-week-old male Wistar rats, that is, in the phase of life with the lowest SD threshold, [K^+^]_o_ must rise to at least ~12 mM.^195^ Figure 8 and the references cited above^191,192^ clearly show that [K^+^]_o_ in the naïve cortex does not reach the value of 12 mM before the DC negativity of SD, but only when the DC negativity is already in full swing. This does not rule out a role for K^+^ in the reaction/diffusion process of SD in our opinion, but also allows for other interpretations in which K^+^ could play a more secondary role. A number of theories about how the SD propagation process might work were reviewed previously.^6^ This subject deserves further research. A possible therapeutic approach indirectly related to K^+^-induced depolarization is, for example, the blockade of voltage-gated Ca^2+^ channels to inhibit SD.^190,196^

## The reaction–diffusion mechanism of SD as a potential target of treatment: Glutamate

With regard to glutamate, the use of genetically encoded optical glutamate sensors has enabled improved spatiotemporal resolution of transient glutamate levels near the SD wavefront.^193^ The co-regional extracellular glutamate increase did not begin until several seconds after the onset of increase in [K^+^]_o_. Increased extracellular glutamate thus appears to be a consequence rather than a cause of processes at the SD wavefront. Consistent with this, SD induction by brain topical glutamate application in vivo required a concentration of ~15 mM,^197,198^ but the peak concentration of glutamate recorded in otherwise naïve tissue is only 90 µM during SD.^199^ Cortical injection of 1 mM glutamate did not result in SD in vivo, although it led to a microelectrode-recorded increase in the extracellular glutamate concentration to ~250 µM.^200^ In addition, glutamate uptake inhibitors strongly increased the extracellular glutamate concentration, but they did not induce SD in vivo.^200,201^

Recently, single-cell blockade and genetic deletion methods were applied to remove functional NMDAR from individual hippocampal CA1 neurons in order to examine the role of NMDAR in the depolarization mechanism without affecting the propagation of SD.^140^ Neuronal input resistance demonstrated a sharp decline at the start of SD, which was unaffected by blocking NMDAR. Instead, the recovery of both membrane potential and neuronal input resistance during the late phase of SD was facilitated by inhibition of NMDAR, indicating that NMDAR are responsible for sustaining the depolarization. This agrees well with earlier findings that an excitatory phase associated with delayed dendritic ionic dyshomeostasis follows the onset of SD and persists for ~2 min in otherwise normal tissue.^202^ This late excitatory phase coincided with a significant increase in presynaptic glutamate release, evidenced by a transient increase in frequency of spontaneous excitatory postsynaptic potentials (EPSC) and paired-pulse depression of evoked EPSCs. NMDAR activation during this late excitatory phase contributed to the duration of individual neuronal depolarizations and delayed recovery of the negative DC shift. Accordingly, NMDAR inhibitors appear to shorten SD in otherwise normal tissue, until they eventually fail to propagate.^202,203^ This inhibitory effect of NMDAR antagonists on SD propagating in normal, metabolically intact tissue was confirmed in numerous studies.^138,204,205^

However, what is true for so many aspects of SD is also true of SD drug sensitivity: it changes along the SD continuum.^6^ Thus, NMDAR antagonists progressively fail to block SD under elevated baseline [K^+^]_o_^195^ or in increasingly ischemic or hypoxic tissue.^90,121,139,206,207^ Elevated baseline [K^+^]_o_ could also be the reason, or at least one of the reasons, for the decrease in SD sensitivity to NMDAR antagonists in the case of cerebral ischemia or hypoxia, since, in contrast to the situation in a normal, metabolically intact cerebral cortex, cerebral ischemia or hypoxia lead to a clear increase in baseline [K^+^]_o_ prior to the onset of SD (Figure 6).^6,191^

Tanaka, Yamamoto et al. performed extensive analyses of the effects of ionotropic and metabotropic glutamate receptor antagonists, various cation channel blockers, various ionic media, intracellular Ca^2+^ chelation, and inhibitors of Ca^2+^ release from intracellular stores on SD induced by oxygen-glucose deprivation in hippocampal slices.^121,208^ Each factor was individually assessed. These experiments revealed not only whether each antagonist functioned as expected, but also its specific effects on the timing of the onset of SD and the magnitude and duration of depolarization. In addition, their approach provided information on washout and reversibility of the blockers. Both studies concluded that no drug or ion substitution alone was able to prevent SD under severe oxygen-glucose deprivation.

Müller and Somjen then described a cocktail that could prevent SD in hypoxic brain slices and thus possibly delay cell death.^209^ This cocktail contained DNQX (10 µM) to block AMPA/kainate receptors, CPP (10 µM) to block NMDARs, TTX (1 µM) to block voltage-sensitive Na^+^ channels, and Ni^2+^ (2 mM) to block voltage-activated Ca^2+^ channels. The authors used 400 µm thick hippocampal slices from ~6-week-old rats at a temperature of 35.5 °C. Whereas hypoxia resulted in SD after 3.5 min, no SD was observed during 20 min of hypoxia plus cocktail. Whether SD would have occurred beyond 20 min was not investigated. Intracellular recordings in the presence of the cocktail showed a slow but not abrupt neuronal depolarization (consistent with the absence of a negative DC shift in extracellular recordings). In addition, there was no dramatic reduction in input resistance, in contrast to the situation when SD occurs. There was recovery after 10 min of hypoxia and washout of the cocktail, but no recovery after 20 min of hypoxia, although no SD had occurred. The authors concluded that 20 min of hypoxia caused irreversible tissue damage, despite the cocktail and the lack of SD.

In another cocktail approach, a bathing medium containing D-AP5 (50 µM), MK-801 (50 µM), NBQX (25 µM), 7-chlorokynurenate (100 µM), and bicuculline (100 µM) to block NMDA, AMPA, kainate, and GABA_A_ receptors succeeded in preventing the signature of SD on the single neuron level in response to severe energy compromise,^207,210^ whereas the glutamate receptor antagonists alone were not sufficient.^211^ In these experiments, ischemia was simulated by replacing 10 mM glucose with 7 mM sucrose, bubbling with 95% N_2_/5% CO_2_ and, to get a reproducible fast onset to the ischemic response, adding 2 mM Na-iodoacetate and 1 mM Na-cyanide to block glycolysis and oxidative phosphorylation. Submerged, 225–300 µm thick slices from 12 to 20 days old rats were kept at 33 °C in these studies.

The efficacy of this combination approach was recently confirmed by an independent group using anoxia in interface slices.^212^ Specifically, their cocktail contained DNQX (50–100 μM) to block AMPA/kainate receptors, MK-801 (50–100 μM), and APV (50–100 μM) to block NMDAR, and bicuculline (25–50 μM) to block GABA_A_ receptors. The slices were taken from 17 to 24 days old rats, were 350 µm thick and held at 36 °C. In some experiments, the blockade of SD was measured with a sharp intracellular electrode and in others with an extracellular electrode recording the DC potential. These recordings confirmed that CsCl (replacing KCl) and QX-314 to suppress voltage-gated K^+^ and Na^+^ currents in the patch pipettes in the experiments by the Attwell group was not the decisive factor for blocking the SD.

The concentrations of inhibitors in these cocktails are so high that it is difficult to imagine how they could be achieved in a patient’s brain.^212^ Even if such concentrations could be achieved, it is highly unlikely that ischemic cell death could be completely prevented by such approaches, since all body cells, that is, even those that do not undergo SD before death, ultimately die when oxidative substrates are completely withdrawn. Nevertheless, it would be useful to systematically investigate whether ischemic cell death could potentially be delayed by a suitable cocktail, that is, whether ischemic tolerance could be increased or not.

## Conclusions

SD is a universal response of the brain to acute stressors, including ischemia, that has been phylogentically conserved from invertebrates to man. In mammals, SD is usually the last potentially reversible phase of life before ischemic cell death occurs. Depending on the context, SD can exhibit both adaptive properties, such as the normal hemodynamic response and cellular regeneration, and maladaptive properties, such as the inverse hemodynamic response and the prolonged intracellular surge of Na^+^ and Ca^2+^ (Figure 1(b)). While the mere occurrence of SD has little correlation with neuronal damage, repetitive episodes and, in particular, prolonged durations of SD signal developing acute lesions. Typically, the longer the local duration of SD, the greater the odds that the tissue will perish. Moreover, decades of evidence, including now clinical data, suggest a direct causative role of SD in this relationship and thus make SD a compelling therapeutic target for neuroprotection. One might paraphrase the insight from Paracelsus regarding pharmaceuticals that “the dose alone makes the poison” (Latin: dosis sola facit venenum). With regard to SD, “the duration alone makes the poison” (Latin: tempus solum facit venenum).
